# Effect of Raspberry (*Rubus indeaus* L.) Juice Fermented by *Limosilactobacillus fermentum* FUA033 on the Human Gut Microbiota Cultured In Vitro: A Multi-Omics Approach

**DOI:** 10.3390/foods14101796

**Published:** 2025-05-18

**Authors:** Ziyan Hua, Yunfan Lv, Han Zhang, Tianyi Mao, Ruyu Xv, Mingxuan Pan, Yadong Hu, Shu Liu, Yaowei Fang

**Affiliations:** 1 Co-Innovation Center of Jiangsu Marine Bio-Industry Technology, Jiangsu Ocean University, Lianyungang 222005, China; 13861820800@163.com (Z.H.); 19516920486@163.com (H.Z.); a1584732025@163.com (T.M.); 2 Jiangsu Key Laboratory of Marine Bioresources and Environment, School of Food Science and Engineering, Jiangsu Ocean University, Lianyungang 222005, China; 15928116880@163.com; 3 Jiangsu Key Laboratory of Marine Biotechnology, School of Food Science and Engineering, Jiangsu Ocean University, Lianyungang 222005, China; 4 Key Laboratory of Human Functional Genomics of Jiangsu Province, Department of Biochemistry and Molecular Biology, Nanjing Medical University, Nanjing 210029, China; yunfan_lv@163.com; 5 Jiangsu Innovation Center of Marine Bioresources, Jiangsu Coast Development Investment Co., Ltd., Nanjing 210019, China; panmingxuan@jsyhkf.com (M.P.); huyadong@jsyhkf.com (Y.H.)

**Keywords:** metabolomics, gut microbiota, *Limosilactobacillus fermentum* FUA033, fermentation, short-chain fatty acids

## Abstract

The gut microbiota plays important functions in human health and influences immune responses, metabolic processes, and several physiological activities. The modulation of the gut microbiota through dietary interventions has emerged as a promising approach, leading to significant interest in the development of functional foods that provide health benefits. In this context, our study investigated the effects of raspberry juice fermented by *Limosilactobacillus fermentum* FUA033 on the structure and metabolism of the gut microbiota. We performed 16S rRNA gene sequencing and nontargeted metabolomics analyses to evaluate changes in the microbial composition and metabolite profiles resulting from fermentation. Our findings revealed that fermented raspberry juice considerably increased the gut microbial diversity and promoted the abundance of beneficial genera. Fermentation substantially increased the production of short-chain fatty acids, such as acetate and butyrate, which increased from 30.09 ± 5.23 mmol/L to 43.07 ± 3.31 mmol/L, and from 7.72 ± 1.72 mmol/L to 15.01 ± 1.26 mmol/L, respectively. Metabolomic analyses also showed significant enhancements in amino acid metabolism pathways, particularly those involving tyrosine, arginine, and proline. These results highlight the potential of fermented raspberry juice as a functional food to improve gut health and metabolic functions.

## 1. Introduction

The human gut contains a complex ecosystem of microorganisms encompassing bacteria, viruses, and eukaryotes (e.g., fungi and protists), which collectively engage in dynamic cross-kingdom interactions that are necessary for host physiology [1]. This microbial consortium plays key roles in immune modulation, metabolic regulation, and disease pathogenesis through intricate host–microbiota crosstalk [2]. Microbial metabolites play important roles in host pathophysiology. For example, short-chain fatty acids (SCFAs; acetate, propionate, and butyrate), trimethylamine N-oxide, and secondary bile acids are linked to metabolic dysfunctions and play important roles in maintaining gut and mental health [3,4,5]. Unlike the host genome, the microbiome exhibits remarkable plasticity, allowing it to adapt dynamically to different types of environmental and host-derived stimuli. Among these environmental factors, diet plays a key role in shaping the gut bacterial composition and gene expression, making it a valuable target for therapeutic manipulation [6].

As a strong association exists between diet and the gut microbiota, dietary interventions may serve as viable alternatives to pharmacological treatments. Dietary components modulate the composition and metabolic activity of the gut microbiota. Some studies have found that a diet rich in fiber promotes microbial diversity, contributing to various benefits, including a decrease in low-density lipoprotein cholesterol, an increase in stool bulk, and an improvement in intestinal transit time [7,8]. Moreover, broccoli sprout extract was found to increase the abundance of butyrate-producing bacteria (e.g., *Faecalibacterium* and *Anaerostipes caccae*), thus improving blood glucose regulation in prediabetic individuals [9].

Among these vegetables and fruits, raspberries are notable for their high polyphenolic content, particularly anthocyanin and ellagitannin. About 75% of the antioxidant activity observed in raspberries can be attributed to their high levels of anthocyanin, ellagitannin (ETs), and ellagic acid (EA) content [10], which impart multiple health benefits through their bioactive properties. Anthocyanins are predominantly found in glycosylated forms such as glucosides and rutinosides. Moreover, ETs undergo microbial metabolism in the gut, where they produce urolithins, which have antioxidant and anti-inflammatory properties [11,12]. Zogona et al. reported that raspberry supplementation alleviates alcohol-induced liver injury, which is closely associated with alterations in the gut microbiota and intestinal barrier dysfunction in mice [13]. Treatment with raspberry polyphenolic extract affects the regulation of liver lipid metabolism and inflammation caused by a high-fat diet [14]. Gut bacteria can be modulated through the consumption of fermented fruits and vegetables. For example, gac fruit juice can increase microbial species richness and promote the growth of Akkermansia. Fermented gac juice promotes the production of SCFAs, which regulate lipid metabolism, suppress inflammation, and modulate energy homeostasis [15,16,17].

In another study, we found that *Limosilactobacillus fermentum* FUA033-fermented raspberry juice enhances the bioaccessibility of raspberry polyphenols by facilitating the conversion of EA into urolithin A, a metabolite with superior bioactivity, including improved antioxidant properties and muscle health [18,19]. Although researchers have determined the health-promoting properties of raspberry polyphenols and the emerging field of fermented functional foods, studies on the mechanisms by which probiotic-fermented raspberry products alter the gut microbiota composition and metabolic outputs are limited. Based on the probiotic potential of *L*. *fermentum* FUA033 in the fermentation of raspberry juice, we established an in vitro fermentation model to investigate the effects of raspberry juice and its fermentation by *L*. *fermentum* FUA033.

In this study, we used a nontargeted metabolomics approach combined with 16S rRNA sequencing to elucidate the gut bacterial composition and metabolic alterations. Additionally, SCFAs were quantified to more comprehensively understand the health benefits of fermented raspberry juice. Our findings provided valuable insights into the effect of fermented raspberry juice on the gut and metabolic health, providing robust evidence for the development of nutritionally beneficial and flavorful fermented raspberry products.

## 2. Materials and Methods

### 2.1. Material

Raspberry samples were purchased from a local fruit market in Lianyungang, Jiangsu Province, China. The geographical coordinates of the sampling location are approximately 119.19° E, 34.50° N, and the samples were stored at −20 °C after they were delivered to the laboratory. The probiotic strain *L. fermentum* FUA033 used in this study was provided by Jiangsu Ocean University (Lianyungang, China).

### 2.2. Raspberry Juice Preparation and Its Probiotic Fermentation

The raspberry juice was prepared following a previously described method [18]. Initially, the juice was extracted using a food-grade pulp separator. The extracted juice was centrifuged at 4000× *g* for 5 min at 4 °C, and the resulting supernatant was collected. For fermentation, the juice was pasteurized at 80 °C for 15 min. The *L. fermentum* FUA033 cells, previously stored at −80 °C, were revived and cultured until they reached an optimal cell density of 5 × 10^7^ CFU/mL. The activated bacterial culture was inoculated separately into pasteurized raspberry juice at a volume ratio of 2% (*v*/*v*) in 100 mL flasks. All juices were fermented independently at 37 °C for 48 h.

### 2.3. Fecal Donors

Healthy volunteers (two females and three males; aged 20–26 years, with BMI ranging from 18.5 to 24.8 kg/m^2^) were recruited based on the following criteria: no antibiotic use within the past three months, no probiotic supplementation within the past month, and no history of gastrointestinal diseases. Each fecal sample was collected and analyzed individually. All participants provided informed consent prior to sample collection. Ethical approval for this study was obtained from Jiangsu Ocean University (Approval No. Jou20241218).

### 2.4. In Vitro Fermentation

The in vitro fermentation experiment was conducted according to a previous study [20]. Unfermented raspberry juice (UFRP) and fermented raspberry juice (FRP) groups were prepared by diluting 1 mL of juice with 9 mL of sterile saline solution, resulting in a 10% (*v*/*v*) juice solution. Wilkins-Chalgren anaerobic medium (WAM) was used as the basal medium. In the experimental groups, 50 mL of WAM was inoculated with 1% (*v*/*v*) fecal homogenate and 1% (*v*/*v*) diluted raspberry juice (fermented or unfermented). The control (Con) group consisted of 50 mL of WAM inoculated with only 1% (*v*/*v*) fecal homogenate. All samples underwent anaerobic incubation at 37 °C for 48 h under an atmosphere composed of N_2_, H_2_, and CO_2_ (80:10:10, *v*/*v*/*v*). After fermentation, the samples were collected and centrifuged for subsequent analyses.

### 2.5. Determination of SCFAs

The SCFA contents were assayed by GC-MS according to a previous study [21]. All standard compounds were obtained from CNW (Beijing, China) or Aladdin (Shanghai, China). Stock solutions of the standards were prepared at 1 mg/mL in methyl tert-butyl ether (MTBE) and stored at −20 °C. Prior to analysis, stock solutions were diluted with MTBE to obtain working solutions. All samples were thawed and homogenized for 3 min. A 50 μL aliquot of each sample was mixed with 100 μL of 0.5% phosphoric acid (*v*/*v*), followed by vortexing for another 3 min. Then, 750 μL of MTBE containing an internal standard was added for extraction. The mixture was vortexed again for 3 min and ultrasonicated for 5 min. After centrifugation at 12,000 r/min for 10 min at 4 °C, the supernatants were collected for SCFA analysis via GC-MS/MS. The samples were analyzed using an Agilent 7890B gas chromatograph coupled with a 7000D mass spectrometer (Agilent Technologies, Santa Clara, CA, USA), equipped with a DB-FFAP capillary column (30 m length × 0.25 mm i.d. × 0.25 μm film thickness, J&W Scientific, Santa Clara, CA, USA). Helium was used as the carrier gas at a flow rate of 1.2 mL/min, with a split injection ratio of 5:1. The oven temperature was initially set at 50 °C for 1 min, increased to 220 °C at a rate of 18 °C/min, and held for 5 min. The injector inlet and transfer line temperatures were 250 °C and 230 °C, respectively. All samples were analyzed in multiple reaction monitoring (MRM) mode. SCFAs were identified by comparison with the Metware Database, a compound library constructed from standard chemical compounds.

### 2.6. 16S rRNA Gene Sequencing and Data Analysis

All fecal samples were stored at −80 °C before genomic DNA extraction with the CTAB method. Bacterial communities were assessed by amplifying the V3–V4 region of the 16S rRNA gene using primer pair 341F (5′-CCTAYGGGRBGCASCAG-3′) and 806R (5′-GGACTACNNGGGTATCTAAT-3′), yielding an amplicon of approximately 470 bp. PCR reactions (total volume 30 µL) contained 15 µL Phusion^®^ High-Fidelity PCR Master Mix (New England Biolabs, Ipswich, MA, USA), 0.2 µM of each primer, and about 10 ng of template DNA. Thermal cycling comprised an initial denaturation at 98 °C for 1 min; 30 cycles of 98 °C for 10 s, 50 °C for 30 s, and 72 °C for 30 s; followed by a final extension at 72 °C for 5 min. Five biological replicates were analyzed for each treatment group (Con, UFRP, FRP; *n* = 5/group). Amplicons were purified with a Qiagen Gel Extraction Kit, quantified, pooled in equimolar amounts, and used to construct libraries with the TruSeq^®^ DNA PCR-Free Kit (Illumina, San Diego, CA, USA). Library quality was assessed on a Qubit 2.0 Fluorometer and an Agilent 2100 Bioanalyzer (Agilent Technologies, Santa Clara, CA, USA), and sequencing was performed on an Illumina NovaSeq 6000 platform (2 × 250 bp). Raw reads were quality-filtered and denoised in QIIME 2 (v 2023.2), and amplicon sequence variants (ASVs) were inferred with Deblur (v 1.1.1).

### 2.7. Nontargeted Metabolomics

The samples stored at −80 °C were thawed on ice and briefly vortexed. A 50 μL aliquot was extracted with 150 μL of extraction solvent (acetonitrile:methanol = 1:4, *v*/*v*) containing 50 μL of internal-standard solution, then vortexed for 3 min. After centrifugation (12,000 rpm, 10 min, 4 °C), 150 μL of the supernatant was collected, incubated at −20 °C for 30 min, and centrifuged again (12,000 rpm, 3 min, 4 °C). Finally, 120 μL of the supernatant was transferred for LC-MS analysis. LC-MS was performed in both positive- and negative-ion modes on a Waters ACQUITY Premier HSS T3 column (1.8 µm, 2.1 mm × 100 mm) with a mobile-phase gradient of 0.1% formic-acid water (A) and 0.1% formic-acid acetonitrile (B) as follows: 5% → 20% B (0–2 min), 20% → 60% B (2–5 min), 60% → 99% B (5–6 min), 99% B (6–7.5 min), then re-equilibration to 5% B. The column temperature was 40 °C, the flow rate 0.4 mL/min, and the injection volume 4 µL. Mass spectra were acquired on a Q Exactive Orbitrap (full scan m/z 75–1000, 35,000 FWHM; data-dependent MS/MS with stepped collision energies of 30/40/50 eV).

Metabolite identification was accomplished by matching precursor masses, MS/MS spectra, and retention times against four complementary spectral libraries: (i) an in-house high-resolution library constructed from authentic standards, (ii) MeTDNA 2.0, (iii) DB-All (integrating HMDB, KEGG, etc.), and (iv) an AI-prediction library providing in silico MS/MS spectra. Only metabolites with a composite identification score ≥ 0.5 and a QC-sample coefficient of variation (CV) < 0.30 were retained for further analysis.

### 2.8. Data Analyses

Data were visualized and statistical analyses were conducted using RStudio (version 2023.6.1.524). For continuous variables that followed a normal distribution, the results are expressed as the mean ± standard deviation. One-way analysis of variance (ANOVA) was conducted to determine differences among multiple groups, whereas Student’s *t*-test or the Wilcoxon rank-sum test was conducted for pairwise comparisons, contingent on distributional assumptions. When the data did not follow a normal distribution, values were summarized as medians with interquartile ranges, and intergroup comparisons were performed using nonparametric methods. To conduct comparisons across multiple nonnormally distributed groups, the Tukey post hoc test was performed. All differences among and between groups were considered to be statistically significant at *p* < 0.05.

## 3. Results and Discussion

### 3.1. Effects on the Gut Microbiota Structure

**Figure 1:**
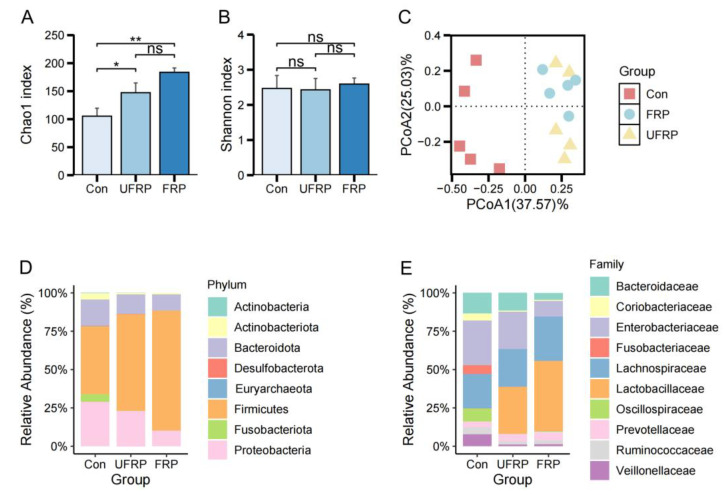
(**A**) Chao1 index and (**B**) Shannon index for Con, UFRP, and FRP. (**C**) Principal coordinate analysis (PCoA) of microbial community structures including Con, FRP, and UFRP. Relative abundance based on ASV at the (**D**) phylum level and (**E**) family level in Con, UFRP, and FRP. Data are presented as mean ± SD. Statistical significance is indicated as: ns, not significant; * *p* < 0.05, ** *p* < 0.01. (*n* = 5/group).

To assess the effect of raspberry and probiotics on the gut microbiota, 16S rRNA gene sequencing was performed. The Chao1 and Shannon indices were analyzed for alpha diversity. The Chao1 index significantly increased from 105.33 ± 14.21 in the Con group to 147.23 ± 17.41 in the UFRP group and 183.68 ± 7.73 in the FRP group (*p* < 0.05), indicating that the components of fermented raspberry juice can promote species richness (Figure 1A). No significant differences were found across the three groups in terms of the Shannon index (Figure 1B). This probably occurred because the changes in species richness did not alter the overall community structure or function. Principal coordinate analysis (PCoA) was performed to evaluate changes in the gut microbial community structure. The Con showed a scattered distribution due to individual differences among the subjects. Compared to Con, FRP resulted in a prominent shift in microbial composition. UFRP presented a similar distribution to that of FRP, suggesting that fermented juice combined with *L. fermentum* FUA033 influenced the structure of the gut microbiota (Figure 1C)*. Firmicutes*, *Bacteroidota*, and *Proteobacteria* were the predominant phyla in all three groups (Figure 1D). The abundance of Firmicutes increased by 33.73% in the FRP group, highlighting the effects of nutrients such as flavonoids and polyphenols in raspberry juice and the cross-feeding of *Firmicutes* by metabolites produced during fermentation [22]. FRP also significantly decreased the abundance of *Fusobacteriota*. Other studies have shown that *Fusobacteriota* may contribute to colorectal cancer, suggesting that adding *L. fermentum* FUA033 may help reduce the abundance of harmful microbes [23].

Further analysis of the microbiota at the family level indicated that the relative abundance of *Prevotellaceae* was significantly increased from 3.76% to 5.65% in the FRP group (Figure 1E). *Prevotellaceae* is associated with a decrease in cholesterol levels and an improvement in glucose metabolism [24]. In the FRP group, the abundance of *Enterobacteriaceae*, which can cause intestinal inflammation and depression, was significantly decreased from 29.11% to 10.16% [25,26]. A recent study suggested that supplementation with *Lactobacillus rhamnosus* and *Lactobacillus murinus* increases butyrate levels and limits nutrient availability, thereby inhibiting the growth of *Enterobacteriaceae* [27]. Additionally, the relative abundance of *Oscillospiraceae* (0.4%) in the FRP group decreased, which is notable, as this family is often enriched in the gut microbiota of patients with colorectal adenocarcinoma [23]. The combined effects of raspberry juice and probiotics reshaped the gut microbiota by enriching beneficial taxa (e.g., *Lactobacillaceae* and *Prevotellaceae*) and reducing pathobionts (e.g., *Enterobacteriaceae*). These findings suggest that fermented raspberry juice may serve as both a prebiotic and probiotic, thereby improving gut health.

### 3.2. Changes in the Microbiota at the Genus Level

**Figure 2:**
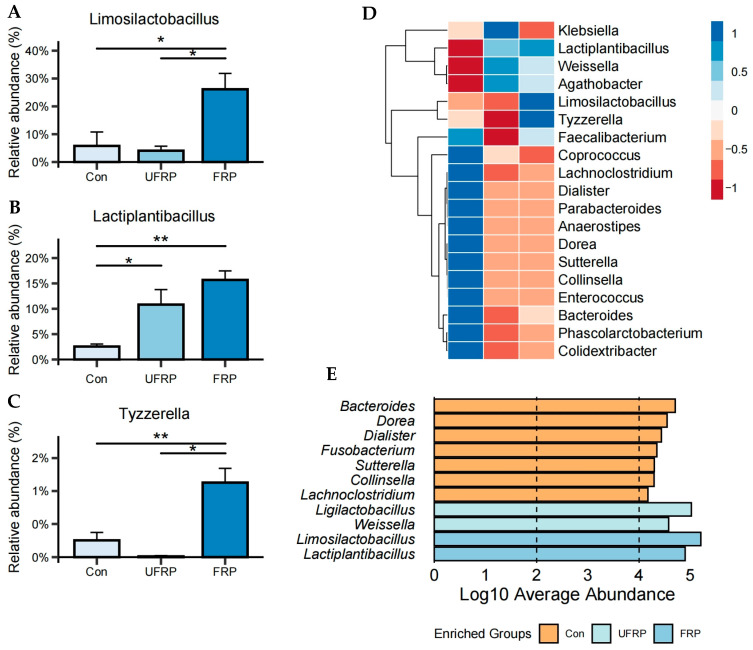
Relative abundance at the genus-level for Con, UFRP, and FRP: (**A**) *Limosilactobacillus*, (**B**) *Lactiplanbacillus*, and (**C**) *Tyzzerella*. (**D**) Relative genus abundance in Con, UFRP, and FRP. (**E**) LDA with effect size measurements showing enriched genus in Con, UFRP, and FRP fecal samples. Data are presented as mean ± SD. Statistical significance is indicated as * *p* < 0.05, ** *p* < 0.01. (*n* = 5/group).

To further investigate the effect of raspberry juice (both fermented and unfermented) on the gut microbiota, a detailed genus-level analysis was performed. Figure 2A shows that the abundance of *Lactiplantibacillus* significantly increased from 2.55 ± 5.01% to 15.69 ± 1.78% in the FRP group (*p* < 0.01). Some studies have reported beneficial effects of *Lactiplantibacillus*, including the enhancement of postprandial lipid metabolism and the relief of chronic constipation in adults [28,29]. This increase in *Lactiplantibacillus* may be attributed to microbial interactions during fermentation, where cross-feeding and metabolic symbiosis occur at the nutritional and metabolomic levels [30]. The abundances of *Limosilactobacillus* and *Tyzzerella* were also significantly increased in the FRP group (*p* < 0.05) (Figure 2B,C). Specifically, the abundance of *Limosilactobacillus* increased from 5.78 ± 5.01% to 26.14 ± 5.7% in the FRP group, suggesting that components in raspberry juice may promote its growth, while the addition of *L. fermentum* FUA033 may play a dominant role in shaping the microbial community. In addition, compared with the Con group, the abundance of *Tyzzerella* in the FRP group increased by approximately 4.4-fold.

The overall genus-level shifts observed in the UFRP and FRP groups are shown in Figure 2D. Both raspberry juice and probiotics contributed to a significant reduction in the abundance of *Enterococcus*, *Colidextribacter*, and *Lachnoclostridium*. *Enterococcus faecalis* can promote liver carcinogenesis under chronic liver disease conditions [31], and its abundance is significantly higher in patients with ulcerative colitis [32]. *Lachnoclostridium* is associated with colorectal adenoma and colon cancer [33], whereas the abundance of *Colidextribacter* is positively correlated with high-fat diets and nonalcoholic fatty liver disease [34]. Linear discriminant analysis effect size (LEfSe) analysis identified key microbial biomarkers across groups (Figure 2E). In the FRP group, *Limosilactobacillus* and *Lactiplantibacillus* were identified as dominant biomarkers, highlighting the microbial shifts driven by raspberry juice and *L. fermentum* FUA033. In the Con group, *Fusobacterium* and *Lachnoclostridium* were identified as biomarkers associated with colorectal cancer and adenoma [23,33]. These findings suggest that the combination of raspberry juice and *L. fermentum* FUA033 can beneficially modulate the gut microbiota, leading to a more favorable microbial profile. It is indicated that sex-related hormone differences may influence the microbiota composition by regulating immune activity. The gut microbiota also participates in sex hormone metabolism via enzymes such as β-glucuronidase [35]. Due to the limited sample size, sex-based differences could not be fully assessed and should be further examined in future studies.

### 3.3. Effects on SCFA Production

**Figure 3:**
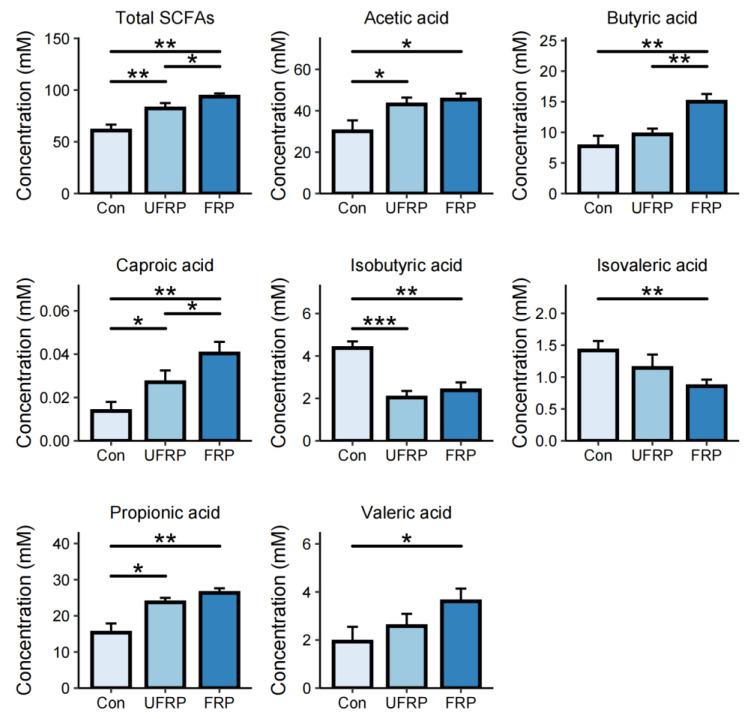
Effects of different treatments on the concentration of short-chain fatty acids (SCFAs). The figure presents the concentration of total SCFAs and individual SCFAs (acetic acid, propionic acid, butyric acid, isobutyric acid, valeric acid, isovaleric acid, and caproic acid) in Con, UFRP, and FRP. Data are presented as mean ± SD. Statistical significance is indicated as * *p* <0.05, ** *p* < 0.01, and *** *p* < 0.001. (*n* = 5/group).

Raspberry represents a comprehensive food matrix as it contains bioactive compounds, dietary fiber, simple sugars, and additional constituents capable of increasing metabolite production by the gut microbiota [36]. Among these microbial metabolites, SCFAs are important for maintaining human physiological health [37]. GC-MS analysis was conducted to quantify the production of SCFAs during in vitro fermentation (Figure 3). The results showed a significant increase in the total concentration of SCFAs from 60.89 ± 5.73 mmol/L in the Con group to 93.68 ± 3.07 mmol/L in the FRP group (*p* < 0.01). In the UFRP group, a comparable increase was also observed, reaching 82.26 ± 5.19 mmol/L (*p* < 0.05), which indicated that raspberry contributes to elevated SCFA levels. Fermentation significantly increased the production of acetic acid, butyric acid, caproic acid, propionic acid, and valeric acid. The concentration of acetic acid increased from 30.09 ± 5.23 mmol/L to 43.07 ± 3.31 mmol/L in the FRP group (*p* < 0.05), and a similar increase was found in the unfermented raspberry group. Butyric acid exhibited an approximately two-fold increase in the FRP group, whereas no significant change was observed in the UFRP group.

This finding indicates that *L. fermentum* FUA033 can produce butyric acid, probably due to specific enzymatic activities involved in the metabolism of raspberry-derived fiber, thereby promoting SCFA production [38]. Short-chain fatty acids play important physiological roles in human metabolism and influence overall health. Acetate, butyrate, and propionate, the three major gut-derived SCFAs, are involved in skeletal muscle metabolism and have strong effects on mucosal and systemic immune responses. A decrease in intestinal SCFA levels is directly linked to an increase in susceptibility to inflammatory and allergic disorders [39,40]. Butyrate plays a key role in repairing gut and mammary epithelial barrier integrity, inhibiting pathogen translocation, and mitigating inflammatory responses [41]. However, in the FRP group, the contents of isobutyric acid and isovaleric acid significantly decreased from 4.37 ± 0.31 mmol/L to 2.38 ± 0.37 mmol/L, and from 1.42 ± 0.15 mmol/L to 0.86 ± 0.10 mmol/L, respectively. Isobutyric acid and isovaleric acid originate from proteolytic fermentation in the human intestine. This decrease may be attributed to the reduced abundance of the major producing genus, Bacteroides [42,43]. The prebiotic effect of raspberry observed in this study is primarily attributed to its rich polyphenol composition, which is further enhanced by the metabolic activity of *L. fermentum* FUA033. Together, they increase SCFA production and gut microbial modulation [44,45].

### 3.4. Effects on Metabolic Profiles

**Figure 4:**
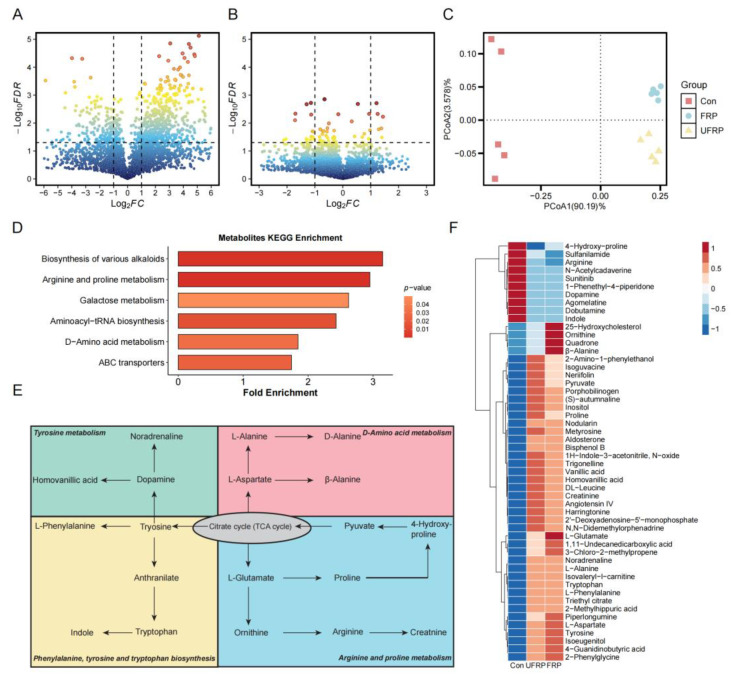
Volcano maps of differential metabolites comparing (**A**) Con and FRP; (**B**) UFRP and FPRP. (**C**) Principal components analysis (PCA) of metabolite structures including Con, FRP, and UFRP. (**D**) KEGG functional pathway of gut microbiota metabolites. (**E**) Metabolic pathway of key compounds. (**F**) Relative metabolite abundance in Con, UFRP, and FRP.

Microbial metabolites act as signaling molecules between the gut microbiota and the host, strongly influencing the physiological health of the host [46]. Nontargeted metabolomics analysis revealed that fermented raspberry juice significantly modulated gut microbial metabolism. Principal component analysis (PCA) results revealed a scattered distribution of metabolite profiles in the Con group, reflecting individual variability, whereas volcano plot analysis revealed distinct metabolomic differences between the Control and FRP groups and between the FRP and UFRP groups (Figure 4A–C). Given the similar metabolite distributions between FRP and UFRP, the following analysis focused on comparing the Control and FRP groups.

The differential metabolites identified through annotation using the Kyoto Encyclopedia of Genes and Genomes (KEGG) database were primarily involved in key metabolic pathways (Figure 4D). Amino acid metabolism dominated the main pathways, including tyrosine metabolism, D-amino acid metabolism, phenylalanine, tyrosine, and tryptophan biosynthesis, as well as arginine and proline metabolism, all of which are interconnected with the tricarboxylic acid (TCA) cycle (Figure 4E,F). In the tyrosine metabolism pathway, increases in noradrenaline and homovanillic acid were observed; elevated noradrenaline levels suggest potential cognitive enhancement and neuroprotective effects, given its important roles in vigilance, attention, learning, and memory [47]. An increase in the levels of homovanillic acid, a microbial-derived metabolite, is associated with improved mental health outcomes through the modulation of synaptic integrity [48]. Additionally, β-alanine concentrations identified in the D-amino acid metabolism pathway imply improvements in physical performance and exercise capacity [49]. The reduction in indole concentrations in the biosynthetic pathways of phenylalanine, tyrosine, and tryptophan may indicate an increase in gut barrier integrity and attenuation of inflammatory responses. In arginine and proline metabolism, an increase in proline and a reduction in arginine levels were detected; elevated proline concentrations are associated with enhanced intestinal homeostasis and therapeutic potential for colitis management [50]. These findings suggest that intervention with fermented raspberry juice and the probiotic *L. fermentum* FUA033 improves host metabolism, potentially enhancing cognitive function, mental health, gut barrier integrity, and physical performance. Further mechanistic studies and clinical investigations are needed to confirm these metabolic effects and their long-term health implications.

### 3.5. Correlations Between the Gut Microbiota Composition and Associated Metabolic Profiles

**Figure 5:**
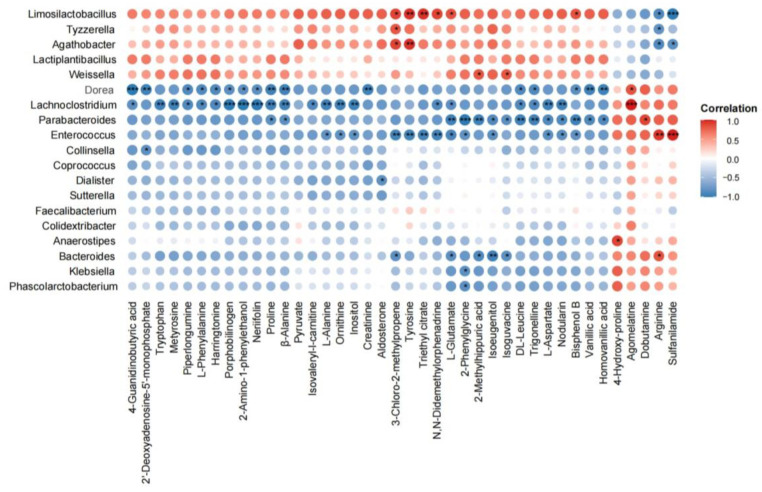
Heatmaps of Spearman’s correlation analysis between differential genera and metabolites. Statistical significance is indicated as * *p* <0.05, ** *p* < 0.01, and *** *p* < 0.001. (*n* = 5/group).

The microbial community, along with metabolomic analysis, was used to evaluate the prebiotic potential of fermented raspberry juice (Figure 5). Notably, *L. fermentum* FUA033 was significantly positively correlated with several metabolites, including 3-chloro-2-methylpropene, tyrosine, trimethyl citrate, and L-glutamate. Tyrosine can be metabolized by the gut microbiota into 4-ethylphenyl sulfate (4EPS), which modulates neural activity and anxiety-like behaviors in mice [51]. Glutamate undergoes extensive recycling between neurons and astrocytes through the glutamate–glutamine cycle, which is required for maintaining brain energy metabolism and supporting glutamatergic neurotransmission [52]. Additionally, *Enterococcus* was positively associated with arginine and sulfanilamide. The reduction in the abundance of *Enterococcus* during fermentation was accompanied by a decrease in arginine levels. Although arginine has beneficial effects in conditions such as diabetes and mitochondrial diseases [53,54], tumor cells accumulate high levels of arginine due to an increase in its uptake and a decrease in its conversion to polyamines. Arginine can act as a second messenger-like molecule, reprogramming cellular metabolism to facilitate tumor growth [55]. In contrast, β-alanine, pyruvate, DL-leucine, and trigonelline were negatively associated with *Dorea* and *Lachnoclostridium*, both of which experienced a decrease in abundance during the fermentation of raspberry juice. These findings suggest that fermentation-induced shifts in microbial composition are closely associated with metabolic changes, which may underlie the health-promoting effects of fermented raspberry juice.

## 4. Conclusions

To summarize, our study showed that raspberry juice fermented by the probiotic strain *L. fermentum* FUA033 effectively modulates the gut microbiota composition, increasing microbial diversity and promoting the growth of beneficial bacterial genera, such as *Lactiplantibacillus* and *Limosilactobacillus*. These microbial changes significantly increase the production of SCFAs, specifically acetate, butyrate, propionate, valeric acid, and caproic acid. The levels of butyrate and acetate, which help maintain intestinal barrier integrity and brain health, increased substantially. The results of the metabolomic analysis further revealed an increase in amino acid metabolism, particularly in the tyrosine, arginine, and proline pathways, highlighting key changes in host metabolic profiles. Importantly, by employing an integrated multi-omics approach, we were able to analyze the interplay between gut microbiota and metabolites at a higher dimension compared to microbiota profiling alone, enabling a more comprehensive understanding of microbiota–metabolite interactions and providing robust validation for the functional properties of the fermented raspberry juice. These findings offer a solid scientific foundation for developing probiotic-fermented raspberry juice as a promising functional food.

Nevertheless, despite demonstrating promising beneficial effects, our current findings based on in vitro fermentation models cannot fully replicate the intricate physiological conditions, host immune responses, and metabolic interactions occurring in vivo. Thus, validation using specific animal models, such as metabolic syndrome, musculoskeletal assessment, and chronic stress models, is necessary to confirm and extend these initial outcomes. Additionally, future studies should evaluate the long-term effects and safety profiles of fermented raspberry juice consumption and include broader populations varying in age, health status, and dietary habits to enhance the robustness and generalizability of functional food research.

## Data Availability

The original contributions presented in this study are included in the article. Further inquiries can be directed to the corresponding authors.
